# Melaminium perchlorate monohydrate

**DOI:** 10.1107/S160053681001857X

**Published:** 2010-05-26

**Authors:** Min Min Zhao, Ping Ping Shi

**Affiliations:** a Ordered Matter Science Research Center, College of Chemistry and Chemical, Engineering, Southeast University, Nanjing 211189, People’s Republic of China

## Abstract

In the title hydrated salt, 2,4,6-triamino-1,3,5-triazin-1-ium perchlorate monohydrate, C_3_H_7_N_6_^+^·ClO_4_^−^·H_2_O, the constituents are linked *via* hydrogen bonds of the O—H⋯O, N—H⋯O, N—H⋯N and N—H⋯Cl types. All the H atoms of the melaminium cation are involved in the hydrogen bonds. The melaminium residues are inter­connected by four N—H⋯N hydrogen bonds, forming chains parallel to (111). The ribbons are inter­connected by other hydrogen bonds as well as by π–π inter­actions [centroid–centroid distance = 3.8097 (7) Å].

## Related literature

For similar organic acid–base compounds, see: Martin & Pinkerton (1995 ▶); Perpétuo & Janczak (2006 ▶). For their  ferroelectric properties, see: Hang *et al.* (2009 ▶), Li *et al.* (2008 ▶). For impedance studies, see; Uthrakumar *et al.* (2008 ▶). 
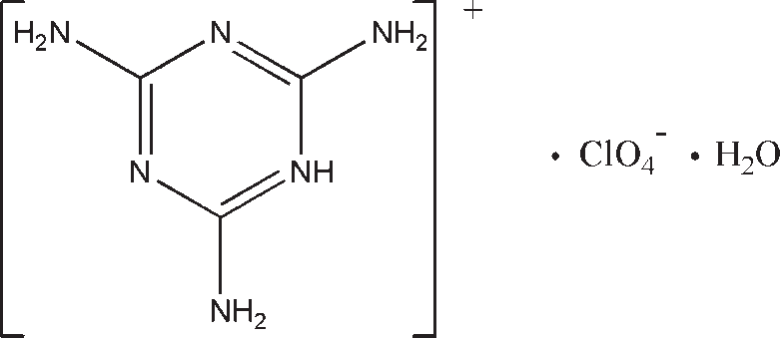


## Experimental

### 

#### Crystal data

C_3_H_7_N_6_^+^·ClO_4_^−^·H_2_O
*M* *_r_* = 244.61
Triclinic, 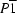

*a* = 5.654 (4) Å
*b* = 7.553 (7) Å
*c* = 11.893 (10) Å
α = 102.72 (4)°
β = 94.58 (3)°
γ = 110.78 (2)°
*V* = 456.1 (7) Å^3^
*Z* = 2
Mo *K*α radiation
μ = 0.44 mm^−1^
*T* = 293 K
0.20 × 0.20 × 0.20 mm

#### Data collection

Rigaku SCXmini diffractometer
Absorption correction: multi-scan (*CrystalClear*; Rigaku, 2005 ▶) *T*_min_ = 0.916, *T*_max_ = 0.916
4902 measured reflections
2051 independent reflections
1719 reflections with *I* > 2σ(*I*)
*R*_int_ = 0.030

#### Refinement

*R*[*F* ^2^ > 2σ(*F* ^2^)] = 0.031
*wR*(*F* ^2^) = 0.091
*S* = 0.90
2051 reflections
163 parameters
10 restraints
H atoms treated by a mixture of independent and constrained refinement
Δρ_max_ = 0.29 e Å^−3^
Δρ_min_ = −0.41 e Å^−3^

### 

Data collection: *CrystalClear* (Rigaku, 2005 ▶); cell refinement: *CrystalClear*; data reduction: *CrystalClear*; program(s) used to solve structure: *SHELXS97* (Sheldrick, 2008 ▶); program(s) used to refine structure: *SHELXL97* (Sheldrick, 2008 ▶); molecular graphics: *SHELXTL* (Sheldrick, 2008 ▶); software used to prepare material for publication: *PRPKAPPA* (Ferguson, 1999 ▶).

## Supplementary Material

Crystal structure: contains datablocks I, global. DOI: 10.1107/S160053681001857X/ng2764sup1.cif

Structure factors: contains datablocks I. DOI: 10.1107/S160053681001857X/ng2764Isup2.hkl

Additional supplementary materials:  crystallographic information; 3D view; checkCIF report

## Figures and Tables

**Table 1:** Hydrogen-bond geometry (Å, °)

*D*—H⋯*A*	*D*—H	H⋯*A*	*D*⋯*A*	*D*—H⋯*A*
O5—H5*B*⋯O4	0.85 (1)	2.16 (1)	2.960 (3)	158 (2)
O5—H5*B*⋯O5^i^	0.85 (1)	2.64 (2)	3.081 (3)	114 (2)
O5—H5*A*⋯O3^ii^	0.84 (1)	2.16 (1)	2.883 (3)	144 (2)
O5—H5*A*⋯O2^iii^	0.84 (1)	2.38 (2)	2.867 (3)	117 (1)
N1—H1*B*⋯O1^iv^	0.86 (1)	2.19 (1)	2.890 (2)	139 (2)
N1—H1*B*⋯O5^iv^	0.86 (1)	2.48 (2)	3.146 (3)	135 (2)
N1—H1*A*⋯N6^v^	0.86 (1)	2.14 (1)	2.998 (3)	178 (2)
N2—H2*B*⋯O4^ii^	0.86 (1)	2.31 (1)	3.086 (3)	151 (2)
N2—H2*B*⋯O2^vi^	0.86 (1)	2.56 (2)	3.108 (3)	123 (2)
N2—H2*A*⋯O2^iii^	0.86 (1)	2.20 (1)	2.979 (3)	150 (2)
N2—H2*A*⋯Cl1^iii^	0.86 (1)	2.99 (1)	3.792 (3)	157 (2)
N3—H3*B*⋯N5^vii^	0.85 (1)	2.23 (1)	3.084 (3)	173 (2)
N3—H3*A*⋯O1^viii^	0.86 (1)	2.19 (1)	3.029 (3)	168 (2)
N4—H4*A*⋯O5^iv^	0.84 (1)	1.90 (1)	2.723 (2)	168 (2)

Symmetry codes: (i) 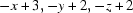; (ii) 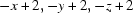; (iii) 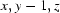; (iv) 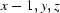; (v) 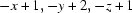; (vi) 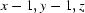; (vii) 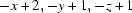; (viii) 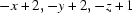.

**Fig. 1.**
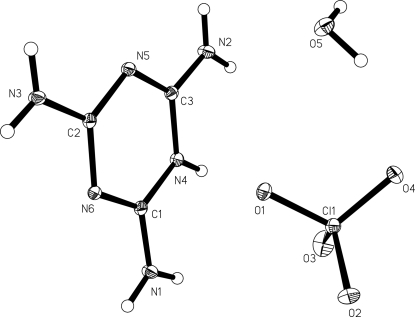
The molecular structure of the title compound, with the atomic numbering scheme. Displacement ellipsoids are drawn at the 30% probability level.

**Fig. 2.**
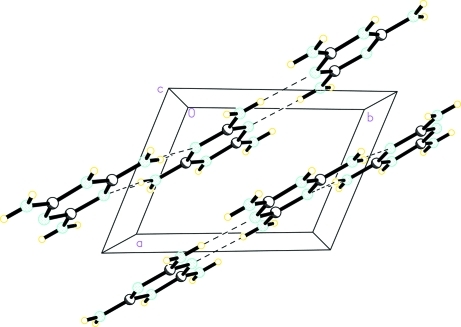
A view of the packing of the title compound, stacking along the c axis. Dashed lines indicate hydrogen bonds.

## supplementary crystallographic information

### Comment

The melamine molecule and its organic and inorganic complexes or salts were widely researched by ancient Chemists (Martin *et al.* 1995; Perpétuo & Janczak, 2006). This study is a part of systematic investigation of dielectric ferroelectric materials, including organic ligands (Li *et al.*, 2008), metal-organic coordination compounds (Hang *et al.*, 2009) and organic inorganic hybrid. Melaminium monoperchlorate monohydrate has no dielectric disuniform from 90 K to 430 K, (m.p. > 470 K).

The asymmetric unit of the title compound is composed of cationic (C_3_H_7_N_6_^+^), anionic (ClO_4_^-^) and one dissociative water molecular(Fig 1). The melaminium cation is protonated at only one melamine ring N atom. The six-membered aromatic ring of melaminium residues exhibit distortions from the regular hexagonal form. The internal C—N—C angle at the protonated N atom(119.41 (14) °) is greater than the other two C—N—C angles of the ring(115.45 (14) ° and 115.48 (14) °) and the internal N—C—N angles involving the nonprotonated ring N atoms (126.09 (15) °) are obviously greater than those containing protonated and non-protonated N atoms(121.65 (15) ° and 121.88 (15) °).

Fig. 2 shows a view down the *c* axis. The melaminium cations are interconnected by four N—H···N hydrogen bonds, forming ribbons parallel to (1 1 1). The ribbons are interconnected by other hydrogen bonds as well as by π-electron ring - π-electron ring interactions with the distance between the centroids of the neighbour melaminium rings (1-*x*,1-*y*,1-*z*) equal to 3.8097 Å. Melamine and its derivatives and organic and inorganic complexes or salts can develop well defined non-covalent supramolecular architectures *via* multiple hydrogen bonds. The hydrogen bonds are summarized in Tab. 1. The H atom of the protonated ring N atom (H4a) is donated to the water molecule, being involved in a strong N—H···O hydrogen bond. The other amine H atoms are involved in N—H···O, N—H···N and N—H···Cl hydrogen bonds. ClO_4_^-^ anions take part in electrostatics equilibrium with the melaminium cations. They are also involved in N—H···O, O—H···O, and N—H···Cl hydrogen bonds.

### Experimental

Single crystals of melaminium monoperchlorate monohydrate are prepared by slow evaporation at room temperature of an water solution of melamine and perchloric acid.

Dielectric studies (capacitance and dielectric loss measurements) were performed on powder samples which have been pressed into tablets on the surfaces of which a conducting carbon glue was deposited. The automatic impedance TongHui2828 Analyzer has been used (Uthrakumar *et al.*, 2008). In the measured temperature ranges (90 K to 450 K, m.p. > 470 K), the title structure showed no dielectric disuniform.

### Refinement

All the hydrogens were discernible in the difference electron density maps. The positions of the H atoms of the melamine cations were refined using a riding model with N—H = 0.86 Å and *U*_iso_(H) = 1.2*U*_eq_(N). The coordinates if the water hydrogens have been refined under restrains 0.84 Å; *U*_iso_(H) = 1.2*U*_eq_(O). (The constrained and the restrained values fit well to the trial refinement with the freely refined hydrogen parameters.)

### Crystal data

**Table:**

C_3_H_7_N_6_^+^·ClO_4_^−^·H_2_O	*Z* = 2
*M**_r_* = 244.61	*F*(000) = 252
Triclinic, *P*1	*D*_x_ = 1.781 Mg m^−^^3^
Hall symbol: -P 1	Mo *K*α radiation, λ = 0.71073 Å
*a* = 5.654 (4) Å	Cell parameters from 1388 reflections
*b* = 7.553 (7) Å	θ = 3.0–27.6°
*c* = 11.893 (10) Å	µ = 0.44 mm^−^^1^
α = 102.72 (4)°	*T* = 293 K
β = 94.58 (3)°	Prism, colorless
γ = 110.78 (2)°	0.20 × 0.20 × 0.20 mm
*V* = 456.1 (7) Å^3^	

### Data collection

**Table:**

Rigaku SCXmini diffractometer	2051 independent reflections
Radiation source: fine-focus sealed tube	1719 reflections with *I* > 2σ(*I*)
graphite	*R*_int_ = 0.030
Detector resolution: 28.5714 pixels mm^-1^	θ_max_ = 27.5°, θ_min_ = 3.0°
CCD_Profile_fitting scans	*h* = −7→7
Absorption correction: multi-scan (*CrystalClear*; Rigaku, 2005)	*k* = −9→9
*T*_min_ = 0.916, *T*_max_ = 0.916	*l* = −15→14
4902 measured reflections	

### Refinement

**Table:**

Refinement on *F*^2^	Primary atom site location: structure-invariant direct methods
Least-squares matrix: full	Secondary atom site location: difference Fourier map
*R*[*F*^2^ > 2σ(*F*^2^)] = 0.031	Hydrogen site location: inferred from neighbouring sites
*wR*(*F*^2^) = 0.091	H atoms treated by a mixture of independent and constrained refinement
*S* = 0.90	*w* = 1/[σ^2^(*F*_o_^2^) + (0.0659*P*)^2^ + 0.0106*P*] where *P* = (*F*_o_^2^ + 2*F*_c_^2^)/3
2051 reflections	(Δ/σ)_max_ = 0.001
163 parameters	Δρ_max_ = 0.29 e Å^−^^3^
10 restraints	Δρ_min_ = −0.41 e Å^−^^3^

### Special details

**Table:**

Geometry. All esds (except the esd in the dihedral angle between two l.s. planes) are estimated using the full covariance matrix. The cell esds are taken into account individually in the estimation of esds in distances, angles and torsion angles; correlations between esds in cell parameters are only used when they are defined by crystal symmetry. An approximate (isotropic) treatment of cell esds is used for estimating esds involving l.s. planes.
Refinement. Refinement of *F*^2^ against ALL reflections. The weighted *R*-factor wR and goodness of fit *S* are based on *F*^2^, conventional *R*-factors *R* are based on *F*, with *F* set to zero for negative *F*^2^. The threshold expression of *F*^2^ > σ(*F*^2^) is used only for calculating *R*-factors(gt) etc. and is not relevant to the choice of reflections for refinement. *R*-factors based on *F*^2^ are statistically about twice as large as those based on *F*, and *R*- factors based on ALL data will be even larger.

### Fractional atomic coordinates and isotropic or equivalent isotropic displacement parameters (Å^2^)

**Table:**

	*x*	*y*	*z*	*U*_iso_*/*U*_eq_	
C1	0.5601 (3)	0.8552 (2)	0.63004 (14)	0.0133 (3)	
C2	0.7940 (3)	0.7067 (2)	0.53001 (14)	0.0129 (3)	
C3	0.7094 (3)	0.6425 (2)	0.70349 (14)	0.0130 (3)	
O1	1.0855 (2)	1.12497 (17)	0.75979 (9)	0.0166 (3)	
O2	1.1733 (2)	1.45744 (17)	0.84904 (11)	0.0201 (3)	
O3	0.8606 (3)	1.2106 (2)	0.90661 (11)	0.0274 (3)	
O4	1.2993 (3)	1.26696 (19)	0.95543 (11)	0.0244 (3)	
O5	1.3200 (3)	0.87257 (18)	0.88354 (10)	0.0196 (3)	
H5B	1.346 (4)	0.9895 (10)	0.9193 (14)	0.023*	
H5A	1.259 (4)	0.7975 (19)	0.9260 (13)	0.023*	
Cl1	1.10439 (7)	1.26476 (5)	0.86874 (3)	0.01324 (13)	
N1	0.4347 (3)	0.9749 (2)	0.64603 (13)	0.0172 (3)	
H1B	0.357 (3)	0.990 (3)	0.7040 (12)	0.021*	
H1A	0.409 (4)	1.034 (3)	0.5952 (13)	0.021*	
N2	0.7213 (3)	0.5547 (2)	0.78757 (13)	0.0169 (3)	
H2B	0.657 (4)	0.576 (3)	0.8500 (11)	0.020*	
H2A	0.818 (3)	0.489 (3)	0.7840 (16)	0.020*	
N3	0.8987 (3)	0.6748 (2)	0.43553 (13)	0.0162 (3)	
H3B	0.975 (3)	0.594 (2)	0.4283 (17)	0.019*	
H3A	0.890 (4)	0.741 (3)	0.3865 (13)	0.019*	
N4	0.5745 (3)	0.7610 (2)	0.71408 (11)	0.0131 (3)	
H4A	0.500 (3)	0.784 (3)	0.7707 (11)	0.016*	
N5	0.8221 (3)	0.61161 (19)	0.61130 (11)	0.0131 (3)	
N6	0.6663 (3)	0.8296 (2)	0.53570 (11)	0.0129 (3)	

### Atomic displacement parameters (Å^2^)

**Table:**

	*U*^11^	*U*^22^	*U*^33^	*U*^12^	*U*^13^	*U*^23^
C1	0.0126 (7)	0.0127 (7)	0.0130 (8)	0.0043 (6)	0.0003 (6)	0.0015 (6)
C2	0.0130 (7)	0.0121 (7)	0.0118 (8)	0.0041 (6)	0.0006 (6)	0.0012 (6)
C3	0.0115 (7)	0.0123 (7)	0.0136 (8)	0.0036 (6)	0.0014 (6)	0.0024 (6)
O1	0.0217 (6)	0.0173 (6)	0.0111 (6)	0.0095 (5)	0.0030 (5)	0.0008 (5)
O2	0.0241 (7)	0.0143 (6)	0.0249 (7)	0.0091 (5)	0.0061 (5)	0.0073 (5)
O3	0.0210 (7)	0.0342 (8)	0.0240 (7)	0.0048 (6)	0.0153 (6)	0.0072 (6)
O4	0.0325 (8)	0.0254 (7)	0.0162 (6)	0.0170 (6)	−0.0064 (5)	0.0012 (5)
O5	0.0282 (7)	0.0153 (6)	0.0185 (6)	0.0093 (5)	0.0111 (5)	0.0067 (5)
Cl1	0.0151 (2)	0.0145 (2)	0.0113 (2)	0.00664 (15)	0.00399 (14)	0.00364 (15)
N1	0.0233 (8)	0.0230 (8)	0.0138 (7)	0.0167 (6)	0.0076 (6)	0.0069 (6)
N2	0.0202 (8)	0.0240 (8)	0.0151 (7)	0.0145 (6)	0.0089 (6)	0.0099 (6)
N3	0.0240 (8)	0.0194 (7)	0.0136 (7)	0.0151 (6)	0.0082 (6)	0.0077 (6)
N4	0.0151 (7)	0.0163 (7)	0.0102 (7)	0.0079 (6)	0.0060 (5)	0.0039 (6)
N5	0.0154 (7)	0.0146 (7)	0.0119 (7)	0.0079 (6)	0.0040 (5)	0.0044 (5)
N6	0.0159 (7)	0.0145 (6)	0.0103 (7)	0.0084 (6)	0.0028 (5)	0.0029 (5)

### Geometric parameters (Å, °)

**Table:**

C1—N6	1.324 (2)	O3—Cl1	1.4318 (16)
C1—N1	1.325 (2)	O4—Cl1	1.4406 (16)
C1—N4	1.362 (2)	O5—H5B	0.845 (5)
C2—N3	1.327 (2)	O5—H5A	0.843 (5)
C2—N5	1.356 (2)	N1—H1B	0.855 (5)
C2—N6	1.357 (2)	N1—H1A	0.860 (5)
C3—N2	1.325 (2)	N2—H2B	0.861 (5)
C3—N5	1.329 (2)	N2—H2A	0.859 (5)
C3—N4	1.360 (2)	N3—H3B	0.854 (5)
O1—Cl1	1.4493 (16)	N3—H3A	0.855 (5)
O2—Cl1	1.4446 (18)	N4—H4A	0.840 (5)
			
N6—C1—N1	120.73 (15)	O2—Cl1—O1	108.79 (9)
N6—C1—N4	121.88 (15)	C1—N1—H1B	123.3 (14)
N1—C1—N4	117.39 (15)	C1—N1—H1A	123.2 (14)
N3—C2—N5	116.85 (15)	H1B—N1—H1A	113.2 (19)
N3—C2—N6	117.06 (15)	C3—N2—H2B	122.9 (13)
N5—C2—N6	126.09 (15)	C3—N2—H2A	117.4 (13)
N2—C3—N5	120.63 (15)	H2B—N2—H2A	118.9 (18)
N2—C3—N4	117.70 (15)	C2—N3—H3B	119.0 (13)
N5—C3—N4	121.65 (15)	C2—N3—H3A	116.9 (13)
H5B—O5—H5A	109.5 (11)	H3B—N3—H3A	124.0 (19)
O3—Cl1—O4	110.66 (10)	C3—N4—C1	119.41 (14)
O3—Cl1—O2	109.04 (8)	C3—N4—H4A	124.6 (13)
O4—Cl1—O2	109.67 (9)	C1—N4—H4A	116.0 (13)
O3—Cl1—O1	109.34 (9)	C3—N5—C2	115.48 (14)
O4—Cl1—O1	109.31 (9)	C1—N6—C2	115.45 (14)

### Hydrogen-bond geometry (Å, °)

**Table:**

*D*—H···*A*	*D*—H	H···*A*	*D*···*A*	*D*—H···*A*
O5—H5B···O4	0.85 (1)	2.16 (1)	2.960 (3)	158 (2)
O5—H5B···O5^i^	0.85 (1)	2.64 (2)	3.081 (3)	114 (2)
O5—H5A···O3^ii^	0.84 (1)	2.16 (1)	2.883 (3)	144 (2)
O5—H5A···O2^iii^	0.84 (1)	2.38 (2)	2.867 (3)	117 (1)
N1—H1B···O1^iv^	0.86 (1)	2.19 (1)	2.890 (2)	139 (2)
N1—H1B···O5^iv^	0.86 (1)	2.48 (2)	3.146 (3)	135 (2)
N1—H1A···N6^v^	0.86 (1)	2.14 (1)	2.998 (3)	178 (2)
N2—H2B···O4^ii^	0.86 (1)	2.31 (1)	3.086 (3)	151 (2)
N2—H2B···O2^vi^	0.86 (1)	2.56 (2)	3.108 (3)	123 (2)
N2—H2A···O2^iii^	0.86 (1)	2.20 (1)	2.979 (3)	150 (2)
N2—H2A···Cl1^iii^	0.86 (1)	2.99 (1)	3.792 (3)	157 (2)
N3—H3B···N5^vii^	0.85 (1)	2.23 (1)	3.084 (3)	173 (2)
N3—H3A···O1^viii^	0.86 (1)	2.19 (1)	3.029 (3)	168 (2)
N4—H4A···O5^iv^	0.84 (1)	1.90 (1)	2.723 (2)	168 (2)

Symmetry codes: (i) −*x*+3, −*y*+2, −*z*+2; (ii) −*x*+2, −*y*+2, −*z*+2; (iii) *x*, *y*−1, *z*; (iv) *x*−1, *y*, *z*; (v) −*x*+1, −*y*+2, −*z*+1; (vi) *x*−1, *y*−1, *z*; (vii) −*x*+2, −*y*+1, −*z*+1; (viii) −*x*+2, −*y*+2, −*z*+1.

## References

[bb1] Ferguson, G. (1999). *PRPKAPPA* University of Guelph, Canada.

[bb2] Hang, T., Fu, D. W., Ye, Q. & Xiong, R. G. (2009). *Cryst. Growth Des.* **5**, 2026–2029.

[bb3] Li, X. Z., Qu, Z. R. & Xiong, R. G. (2008). *Chin. J. Chem.* **11**, 1959–1962.

[bb4] Martin, A. & Pinkerton, A. A. (1995). *Acta Cryst.* C**51**, 2174–2177.

[bb5] Perpétuo, G. J. & Janczak, J. (2006). *Acta Cryst.* C**62**, o372–o375. 10.1107/S0108270106015873 16823205

[bb6] Rigaku (2005). *CrystalClear* Rigaku Corporation, Tokyo, Japan.

[bb7] Sheldrick, G. M. (2008). *Acta Cryst.* A**64**, 112–122. 10.1107/S0108767307043930 18156677

[bb8] Uthrakumar, R., Vesta, C., Raj, C. J., Dinakaran, S., Dhas, R. C. & Das, S. J. (2008). *Cryst. Res. Technol.* **43**, 428–432.

